# Reconsidering unconscious persistence: Suppressing unwanted memories reduces their indirect expression in later thoughts

**DOI:** 10.1016/j.cognition.2019.02.016

**Published:** 2019-06

**Authors:** Yingying Wang, Andrea Luppi, Jonathan Fawcett, Michael C. Anderson

**Affiliations:** a Academy for Advanced Interdisciplinary Studies, Peking University, China; b Division of Anaesthesia, Department of Clinical Neurosciences, University of Cambridge, UK; c Department of Psychology, Harvard University, United States; d Department of Psychology, Memorial University of Newfoundland, Canada; e MRC Cognition and Brain Sciences Unit, University of Cambridge, UK

**Keywords:** Retrieval suppression, Inhibition, Implicit memory, Conceptual representation, Think/no-think (TNT), Remote associates test (RAT)

## Abstract

When we seek to forget unwelcome memories, does the suppressed content still exert an unconscious influence on our thoughts? Although intentionally stopping retrieval of a memory reduces later episodic retention for the suppressed trace, it remains unclear the extent to which suppressed content persists in indirectly influencing mental processes. Here we tested whether inhibitory control processes underlying retrieval suppression alter the influence of a memory’s underlying semantic content on later thought. To achieve this, across two experiments, we tested whether suppressing episodic retrieval of to-be-excluded memories reduced the indirect expression of the unwanted content on an apparently unrelated test of problem solving: the remote associates test (RAT). Experiment 1 found that suppressed content was less likely than unsuppressed content to emerge as solutions to RAT problems. Indeed, suppression abolished evidence of conceptual priming, even when participants reported no awareness of the relationship between the memory and the problem solving tasks. Experiment 2 replicated this effect and also found that directing participants to use explicit memory to solve RAT problems eliminated suppression effects. Experiment 2 thus rules out the possibility that suppression effects reflect contamination by covert explicit retrieval strategies. Together, our results indicate that inhibitory control processes underlying retrieval suppression not only disrupt episodic retention, but also reduce the indirect influence of suppressed semantic content during unrelated thought processes. Considered with other recent demonstrations of implicit suppression effects, these findings indicate that historical assumptions about the persisting influence of suppressed thoughts on mental health require closer empirical scrutiny and need to be reconsidered.

## 1 Introduction

Psychologists have long theorized that unwanted memories influence our thoughts outside of our awareness, even after we try to put those memories out of mind. In classical psychoanalytic thought, for example, the indirect influence of memories was a cornerstone process in which repressed contents discreetly re-emerged in patients’ thinking and actions, often to the detriment of their mental health (Freud, 1915, Freud, 1966; see Berlin, 2011). These classical ideas have influenced modern clinical thought (e.g., Pennebaker, 1997, Pennebaker and Susman, 1988, Schwartz, 1990, Westen, 1998) and are well reflected in the concept of experiential avoidance (e.g., Hayes, Wilson, Gifford, Follette, & Strosahl, 1996), in which attempts to avoid awareness of private experiences (e.g. thoughts, memories, emotions) are hypothesized to contribute to psychopathology. A recurring theme among these proposals is the idea that suppressing a thought is ultimately ineffective not only because it is assumed that the memory’s persistence requires sustained and stressful suppression but also because it does little to limit the unconscious effects of that experience on later thought. Key to this view is the proposal that a memory’s indirect influence survives the loss of its conscious retention, a possibility for which there is support (e.g., Mitchell, Kelly, & Brown, 2018) and which receives credibility from dissociations between explicit and implicit memory (Gabrieli, 1998, Schacter et al., 1993). Critically, however, this view further requires those implicit influences of a memory to be immune to the control processes by which people suppress its awareness. If so, then by wilfully forgetting unwanted memories, we ironically surrender control of our thoughts to the pernicious re-emergence of the unwanted content, enabling it to hold sway over mental life.

In this article, we examine whether suppressing unwanted memories preserves their implicit influence on later thought processes. In particular, we focus on whether inhibitory control processes that are engaged when suppressing retrieval of a memory affect the semantic content underlying the trace, and its capacity to broadly influence thought processes, outside of a person’s awareness. We were led to this focus for several reasons. First, growing evidence suggests that inhibitory processes engaged to suppress retrieval of unwanted memories reduce explicit memory for the suppressed events, contributing to a phenomenon known as suppression-induced forgetting (Anderson & Green, 2001; see Anderson & Hanslmayr, 2014 for a review). Notably, difficulties in forgetting via retrieval suppression have been associated with worse mental health status: impaired suppression-induced forgetting has been found in individuals suffering from post-traumatic stress disorder (Catarino, Kupper, Werner-Seidler, Dalgleish, & Anderson, 2015), high ruminators (Fawcett et al., 2015, Hertel et al., 2018), people with higher trait anxiety (Marzi, Regina, & Righi, 2014; see also Benoit, Davies, & Anderson, 2016), and in participants suffering from depression (Hertel and Gerstle, 2003, Noreen and Ridout, 2016a, Noreen and Ridout, 2016b, Zhang et al., 2016). In contrast, better suppression-induced forgetting ability predicts reduced intrusiveness of a traumatic film over a one-week period (Streb, Mecklinger, Anderson, Lass-Hennemann, & Michael, 2016) and also predicts reduced negative affect associated with suppressed content (Gagnepain, Hulbert, & Anderson, 2017). These apparent benefits led us to propose that memory control is a fundamental mechanism of emotion regulation (Engen & Anderson, 2018) and that it may not, after all, leave harmful implicit influences. Second, inhibitory processes engaged during retrieval suppression not only affect episodic memory, but also perceptual implicit memory (Gagnepain et al., 2014, Kim and Yi, 2013); thus, at least for repetition priming of perceptual content, inhibition does not preserve the indirect influence of an experience. Third, retrieval suppression not only down-regulates activity in the hippocampus, a structure associated with explicit memory, but also in neocortical regions likely involved in representing the suppressed content (Benoit et al., 2015, Depue et al., 2007, Gagnepain et al., 2014, Gagnepain et al., 2017). Thus, when suppressing episodic memories, control processes also affect brain regions likely to contribute to implicit memory.

Taken together, these observations suggest that inhibitory processes engaged to suppress unwanted memories may not, in general, leave implicit memory intact. This possibility would have important implications: suppression may disrupt conceptual implicit memory effects that are essential in mediating unconscious influences. As a result, the ideas underlying a suppressed memory may not emerge indirectly to control thoughts and behaviour, as is sometimes assumed. Indeed, several recent findings are consistent with the possibility that intentional retrieval suppression disrupts conceptual implicit memory. It remains unclear, however, whether inhibitory control processes underlying retrieval suppression are responsible for these effects, and whether these effects are truly implicit. We discuss these findings next.

### 1.1 Suppression-Induced forgetting in conceptual implicit memory

Much of the work examining how retrieval suppression affects explicit and implicit memory has used a procedure known as the Think/No-Think (hereinafter, TNT) task (Anderson & Green, 2001). The TNT task requires participants to attend to reminders of previously acquired associations involving those items. For each reminder, they are cued either to retrieve the associated memory (Think trials) or to instead suppress its retrieval (No-Think trials). Repeatedly suppressing retrieval impairs retention of the associated memory on later episodic recall tests (Anderson & Green, 2001; see Anderson & Hanslmayr, 2014, for a review); retrieving memories, in contrast, enhances their later recall. Evidence suggests that retrieval suppression is achieved, in part, by inhibitory control mechanisms that suppress the excluded trace, as evidenced by suppression-induced forgetting that generalizes to multiple reminders, a property known as cue-independence (Anderson & Green, 2001; see Anderson & Hanslmayr, 2014, for a review). The capacity to suppress episodic retrieval and impair memory is achieved by a fronto-parietal control network that is similar to that engaged in stopping prepotent motor actions, and that is engaged irrespective of whether the suppressed events are neutral or negatively valenced, or are verbal or visual in nature (e.g., Anderson et al., 2004, Butler and James, 2010, Depue et al., 2007, Gagnepain et al., 2017; see Anderson & Hanslmayr, 2014, for a review). The apparent generality of this process and its relationship to self-perceived thought control ability (Kupper, Benoit, Dalgleish, & Anderson, 2014) suggest that episodic retrieval suppression may provide a useful model with which to evaluate whether inhibitory processes engaged to suppress unwanted thoughts affect their later indirect influence on cognition.

Several findings using the TNT task already support the idea that retrieval suppression affects conceptual implicit memory. For example, Hertel, Large, Stuck, and Levy (2012) performed a retrieval suppression experiment, using a free-association test to measure suppression effects in implicit memory. Participants associated homographs as cue words (e.g. Straw) with targets (e.g. Hat) that were semantically related to one of the homograph’s meanings. After the Think/No-Think phase, participants performed a free association test, in which they were presented with the very same cue words (e.g. Straw) as prompts. Hertel et al. (2012) found significantly reduced production of (a) targets for No-Think items, compared to Baseline items, and, interestingly, (b) other words in line with the suppressed meaning of the homographic cue (see also Hertel et al., 2018 for a replication of this finding). These findings are consistent with the possibility that suppression engaged inhibition to reduce the accessibility of suppressed concepts, reducing their later indirect influence on the free association task. However, one can also interpret the reduced accessibility of suppressed items in terms of increased interference: by testing free-association using the very same cue word (e.g. Straw) as was used during the No-Think task, recall might have been reduced because the cue word elicited interfering associations that might have been formed during No-think trials, to avoid thoughts of the unwanted targets (Hertel and Calcaterra, 2005, Wang et al., 2015). Thus, although these findings indicate that retrieval suppression can affect performance on a conceptually driven indirect test, the potential role of associative interference in these effects leaves it unclear whether inhibitory processes engaged by retrieval suppression truly affect conceptual implicit memory. Without clarifying this mechanistic ambiguity, it is impossible to know whether effects such as those reported by Hertel et al. (2012) would be specific to tests involving the particular cues used to achieve suppression (as would be true with interference) or would instead generalize to any cues by which putatively inhibited semantics might be accessed (as would be the case with inhibition and cue independence). Only in the latter case could it be accurately said that suppressed ideas were generally less influential on ongoing thought.

Despite the foregoing concerns, two recent findings are less likely to reflect interference processes and suggest that inhibition may indeed affect conceptual implicit memory in retrieval suppression. Taubenfeld, Anderson, and Levy (2018) conducted a retrieval suppression experiment using the Think/No-Think procedure that employed a category verification task as the final measure of indirect influence instead of free association. The category verification task asked participants to judge the truth of categorical statements (e.g., “A sheep is an animal”) as quickly as possible, and reaction times were recorded. Unsurprisingly, categorical statements containing previously studied words were generally verified more quickly, suggesting a conceptual priming benefit for studied items. Critically, when the studied words had been previously suppressed during the TNT task, this conceptual priming effect disappeared, suggesting that suppression had affected implicit memory for the suppressed content. Because this indirect test did not include the originally studied cues, it is more difficult to explain these findings with interference. Unfortunately, because the category verification task presents the previously studied words overtly to participants, participants’ awareness of the relationship between the TNT task and the category verification task likely was quite high, making it difficult to rule out the possibility that suppression effects might arise from how inhibition affects explicit components of the task. Thus, even if inhibitory control is involved in producing this finding, it may not reflect a true impact of inhibition on implicit memory.

A similar ambiguity concerning explicit recognition applies to a related finding reported by Hertel et al. (2018) using flanker interference. Hertel et al. (2018) found that previously suppressed items were significantly less distracting to participants when those suppressed items appeared as flankers during a speeded valence judgment task than when the flankers were instead composed of baseline items that were studied, but not suppressed. Hertel et al. (2018) interpreted this reduced distraction from suppressed items in terms of reduced implicit influence of suppressed concepts on the valence judgment task. However, one cannot be certain that the flanker interference effect in this procedure is not tied to the distracting effects of explicit recognition of the flankers, which may be reduced for suppressed items. Thus, as with the data reported by Taubenfeld et al. (2018), it remains unclear whether the inhibitory processes thought to underlie suppression-induced forgetting truly extend to conceptual implicit memory or whether these findings instead reflect the impact of those processes on explicit memory.

In the current studies, therefore, we sought evidence that suppressing an unwanted memory generally reduces the influence of its semantics on later thought processes, even when associative interference and explicit memory can be persuasively ruled out. Such findings would be consistent with a role of inhibitory control in suppressing general conceptual representations during the process of retrieval suppression.

### 1.2 The current studies

**Fig. 1:**
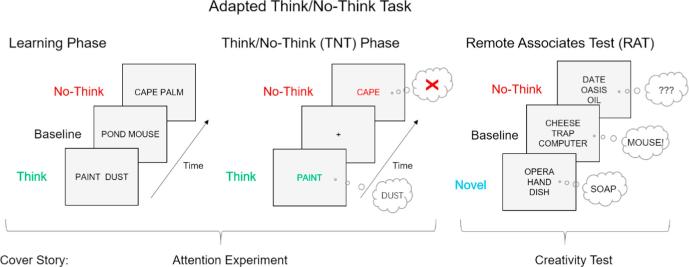
A procedural schematic. In the learning phase, participants encoded cue-target pairs. During the TNT phase, participants suppressed the retrieval of some of the target words (No-think condition: top middle) and recalled others (Think condition: bottom middle). In the Remote Associates Test (i.e., the RAT), participants were asked to generate a word that linked the three cue words provided. Solutions for some of the RAT problems were target words from the studied pairs (i.e., from the No-think and Baseline conditions), but most problems had solutions that were novel words that had not been encoded.

To indirectly measure the impact of episodic retrieval suppression on later cognition with minimal explicit contamination, we replaced the episodic recall test that is typically used at the end of the Think/No-Think procedure with a test first introduced to measure creativity known as the Remote Associates Test (hereinafter referred to as the RAT; Mednick, 1962; see Fig. 1).^1^ The RAT presents participants with simple thought puzzles composed of multiple cue words that all share a hidden idea in common, and participants are asked to identify what that hidden idea is. Specifically, for each puzzle, participants receive three independent cues from mutually distant associative clusters. To solve the problem, participants need to provide a target word that unites all three of the cues. For example, participants might receive OPERA, HAND, and DISH as cues, with the intended solution being SOAP; or they might receive MEASURE, WORM, and VIDEO, with the intended solution TAPE. Solving these puzzles requires a clear focus on the meanings of all three cues, and creative thinking about what they have in common; participants find the task absorbing and they find the moment of insight when they solve the problem enjoyable. Critically, by specially designing RAT problems with answers that appeared as targets in our TNT materials and by administering the TNT task prior to the RAT, we sought to distinguish whether intentionally suppressing episodic retrieval of some items preserved their tendency to emerge spontaneously in later thought, or instead altered it. To ensure that any influence of the TNT materials on the RAT was indirect and spontaneous, we carefully disguised the relationship between the tasks by (a) presenting the tasks as different experiments, (b) ensuring that the RAT cues for each problem had not appeared in the TNT task, and (c) ensuring that few of the RAT problems had solutions from the TNT materials (Gomez-Ariza et al., 2017). Thus, our adaptation of the RAT measures the potential influence of retrieval suppression on an interesting thought process reliant on semantic access (creative problem solving) in a manner that should greatly reduce contamination by explicit memory.

The RAT also provides a useful tool for evaluating the role of inhibition in producing suppression effects in this procedure. Because we designed the RAT cues for each problem to be unassociated to the original study cue for its solution, the cues sidestep the particular cue-target associations used in the original TNT task; as such, any reduced performance observed for suppressed items must reflect a change in accessibility of the suppressed content itself, and not be caused by cue-dependent processes such as associative interference (Anderson and Green, 2001, Anderson and Spellman, 1995). The RAT has already been used to establish cue-independent evidence of conceptually implicit effects in a related memory inhibition phenomenon, retrieval-induced forgetting (Gomez-Ariza et al., 2017). Demonstrating suppression-induced forgetting on our RAT task would extend this finding to the domain of intentional retrieval suppression, and establish the key property of cue-independence, which favors the involvement of inhibitory control as a source of forgetting (Anderson and Bjork, 1994, Anderson and Spellman, 1995). Given the prior evidence for suppression-induced forgetting on perceptually-driven indirect tests, we predicted that suppression would also reduce access to the semantic content underlying the suppressed event, reducing the expression of that semantic content in their problem-solving behaviour.

## 2 Experiment 1: The influence of suppression on problem solving

To test whether suppressed semantic content re-emerges indirectly in later thought processes, we adapted the test phase of the TNT procedure to measure participants’ tendency to generate No-Think, Baseline, and Novel solution words on the RAT (Fig. 1). Each trial of the RAT procedure presented participants with three cue words that were each associated independently to a common solution word based on pre-existing semantic associations (e.g. MEASURE, WORM, and VIDEO for the solution TAPE). The cue words for RAT problems were not encoded during the prior phases and were unassociated to studied cue for its solution, disguising potential relationships between the TNT and RAT tasks. Efforts were made to control participants’ expectations about the relations between the RAT phase and the TNT task. First, the study was introduced as comprising two experiments, which tested attentional control and creative abilities respectively. To make the story more convincing, we told participants that their eye movements would be recorded during the attention test (i.e. TNT phase) and that we were monitoring their ability to stay focused on the cues, despite the demanding cognitive task we had given them (i.e. the TNT task). Second, we implemented measures to reduce the chances that participants would notice links between the critical RAT problems and the earlier studied items, including (a) ensuring that solutions from 60% (36 out of 60) of the RAT problems were novel words that had not been studied and (b) omitting tests of Think items (i.e., items that participants had repeatedly retrieved during the TNT task), the salience of which might “tip subjects off” to the connection between phases (see Gomez-Ariza et al., 2017, for evidence that repeated retrieval practice enhances RAT performance for retrieved items). Finally, as a manipulation check, a post-experimental questionnaire was given to measure whether participants noticed that some of their solutions to the RAT problem overlapped with items they had studied earlier. Taken together, these controls were designed to remove incentives for explicit retrieval in general, and to measure the extent to which it did occur.

### 2.1 Method

#### 2.1.1 Participants

Thirty right-handed native English speakers (20 female participants, mean age = 22.47 years, *SD* = 4.13) participated in exchange for monetary compensation. The sample size was based on conventional sample sizes used in prior work on suppression-induced forgetting, which typically range from 18 to 40.^2^ Participants were recruited from the MRC Cognition and Brain Sciences Unit participant panel and through flyers and online advertisements. They had no reported history of head injury, neurological disease, or learning disability and no red/green color-blindness, and they had not participated in previous studies from the same group before. The project was approved by the Cambridge Psychology Research Ethics Committee, and all participants gave written informed consent.

#### 2.1.2 Materials

The stimuli for the main experiment were drawn from a set of 72 verbal paired associates and 72 RAT problems designed specifically for the current studies. We describe each of these, in turn, and then how they were used to construct materials for a given participant. All paired associates and RAT cues can be found in Appendix A1.

*Paired Associates*. Each word pair was composed of a left-hand word (hereinafter, a cue word) and a right-hand word (hereinafter, a target word). The word pairs for this experiment were made specially so that the target word in each word pair was the solution for one of the RAT problems (e.g., if a RAT solution was TAPE, the paired associate might be NYLON-TAPE). The two words were only weakly associated with each other, as evidenced by low semantic similarity based on the latent semantic indexing (LSI, *M* = 0.070, SD = 0.146) (Deerwester, Dumais, Furnas, Landauer, & Harshman, 1990).

The total list of 72 word-pairs was divided into two subsets, with one set of 36 to be used in the TNT procedure for a given participant. The remaining set of 36 pairs was not studied and provided materials from which to construct RAT problems in the Unprimed condition (to be described shortly). The particular set of 36 that was assigned to the Studied or Unprimed sets was counterbalanced across participants. Each set of 36 pairs was further divided into three lists, which were assigned to the Think, No-Think, and Baseline conditions, when that list served in the Studied condition. The three lists were matched on word frequency, and each appeared equally often in all conditions across participants (see Appendix A2 for a quantitative summary of materials characteristics). Twelve additional word pairs were included as fillers for practice. The word pairs in different subsets were matched on the strength of the associative relation between the cue and target words, word length and frequency of cue words and solution words. The association strength was based on LSI (Deerwester et al., 1990) and word frequency was measured by the University of South Florida word association norms (Nelson, McEvoy, & Schreiber, 2004).

*Remote Associates Test Problems*. To test the accessibility of the target members for each word pair used in the TNT paradigm, we designed a series of matched Remote Associates Test problems. Each test problem was comprised of three cue words with no obvious relationships (e.g., MEASURE, WORM, and VIDEO). Each of the three words was associated with a fourth (unmentioned) word, which was the intended solution for that particular RAT problem (e.g., TAPE). The three cue words were associated with the solution word by means of synonymy, formation of a compound word, or semantic association. Each cue and solution element was always a single word. We constructed a RAT problem for each of the target members from our set of 72 pairs, along with an additional 4 pairs to be used as practice problems. We selected most of the RAT problems from a normative stimulus set (Bowden & Jung-Beeman, 2003); we constructed the remainder by selecting cue words associated to each target word using the University of South Florida word association norms (Nelson et al., 2004).

A given participant was tested on sixty RAT problems, among which 24 were critical ones, the solutions for which were the target items of the word pairs in the No-Think and Baseline conditions. The remaining 36 RAT problems were Unprimed items the solutions for which were studied during the TNT procedure (corresponding to the unused set of 36 word-pairs). The RAT problems in different subsets were matched on the strength of the associative relation between the RAT problems and their solutions, word length and frequency of cue words and solution words (Nelson et al., 2004). The semantic relation between the three cue words in the RAT problems with the cue word in the word pairs was very weak (LSI = 0.009, SD = 0.042) (Deerwester et al., 1990).

#### 2.1.3 Design

We used a within-subject design, with condition manipulated across three levels: Baseline, No-Think, and Novel. The items participating in the Baseline and No-Think conditions were studied initially; afterwards, No-Think items were suppressed during No-Think trials of the TNT task. Items in the Novel condition never were encoded and were thus unprimed when they were probed on the final remote associates test at the end of the experiment. Experiment 1 tested whether suppressing retrieval during No-Think trials reduced the later accessibility of the suppressed words on a conceptual implicit memory task. Our dependent measure was the proportion of RAT problems solved with the intended solution, in each of the conditions.

#### 2.1.4 Procedure

The modified TNT procedure (Anderson & Green, 2001) consisted of three phases: the study phase, the TNT phase, and the RAT phase.

*Study phase*. Participants first studied 48 word-pairs (12 for each condition: Think, No-Think, Baseline, and Filler), each for 4 s (inter-stimulus interval [ISI] = 0.5 s). Test-feedback cycles followed, in which participants were presented with each cue word for up to 3 s and were asked to recall the target word as quickly as possible. The cue word disappeared from the screen upon reporting or when the response window expired. The target word was then given on the screen for 1.5 s and participants were instructed to use the feedback to improve their memory of the pairs. Test-feedback cycles on all the pairs continued up to three times or until a minimum of 60% of the pairs were correctly recalled. A criterion test was then given to the participants, in which each cue word was presented on the screen for up to 2 s and participants sought to report the target word. The criterion test was used to measure which items were correctly memorized during the study phase, so that we could compute final test performance conditional on correct initial learning of a given item. No feedback was given in the criterion test. In the study, test-feedback, and criterion test phases, the order of pair presentation (or pair testing) was determined by blocked randomization: in each consecutive block of 8 trials, 2 (future) Think, No-Think, Baseline, and filler items appeared in random order, to ensure that serial position effects during encoding or training did not differentially influence the memorability of the pairs.

*TNT phase*. Once participants had learnt the word-pairs to criterion, they entered the Think/No-Think phase of the experiment. Participants were told that they would be presented with a subset of the cue words, one at a time. Each presented cue word would appear for 3 s either in green (Think trial) or in red (No-Think trial) font. For Think trials, participants were instructed to recall the associated target word as quickly as possible and to rehearse it silently for the 3 s duration of the trial. For No-Think trials, participants were asked to avoid thinking about the associated target word while sustaining their attention on the cue word for the 3 s trial. Specifically, they were asked to block thoughts of the target word by sensing the urge to retrieve it and then actively suppressing the urge. We used the Direct Suppression variant of the suppression instructions (Benoit and Anderson, 2012, Bergstrom et al., 2009): participants were asked not to replace the target word with any other diversionary thoughts or images, but simply to stop themselves from retrieving the target. To ensure that participants understood these instructions, practice trials using filler items were presented preceding the TNT phase proper, along with a structured feedback questionnaire was used to focus participants on each element of the instructions.

Following practice, participants completed the TNT task using the 24 critical cue words: 12 in the Think condition and 12 in the No-Think condition (the remaining, Baseline items were excluded from this phase). The task was divided into 5 sessions, each with two repetitions of the 24 Think/No-Think items. The study phase and the TNT phase were justified to participants as an attention test – making no mention of any test to follow. Specifically, participants were told that we were measuring their ability to ignore distraction, such as distraction that the target words might cause while focusing their attention on the cue words. To make the story convincing, we told participants that their eye movements would be recorded during the TNT phase with a desktop eye tracker (which we did in Experiment 1, but not in Experiment 2, even though the eye tracker was present). After the TNT phase, participants were told that they had reached the end of the attention experiment.

*RAT phase*. Following the TNT phase, we administered the RAT to measure problem solving for the 24 critical words (i.e. 12 No-Think targets and 12 Baseline targets) as well as the 36 unstudied words serving in the Novel condition. Participants were told that they would be starting a new task that measured their creative ability. The connection between the RAT phase and the prior phases of the experiment were not mentioned. Sixty RAT problems were given to each participant across four blocks. On each trial, the triplet cues of a RAT problem were presented in the center of the screen. Participants were instructed to solve the problem by generating a fourth word that was associated with each of the three cue words. Participants were given up to 60 s for each problem and were encouraged to use as much time as they needed to solve the problem. Four RAT problems, with solutions not corresponding to any of the studied words, were given at the beginning of this phase for practice.

*Post-Experimental Questionnaire*. A post-experimental questionnaire was given after the RAT phase to measure participants’ awareness of the association between the RAT test and the memory tasks. The questionnaire contained four questions (see Appendix C). The first question asked participants whether they had realized that some of the solution words they generated were ones that they had studied in the prior phases (with ratings of 0, 1, or 2, for “Didn’t notice”, “Unsure” and “Noticed”). Questions 2a and 2b were only answered by participants if selected “Unsure” or “Noticed” in question 1. It asked whether, if they had realized the connection between phases, then (a) to what extent did they simply continue to solve problems based on general knowledge (ratings 0–4, ranging from Disagree to Strongly Agree) and (b) to what extent did they try to retrieve answers from the earlier phase (with ratings from 0 to 4, Disagree to Strongly Agree). The final question asked participants about when they noticed some of the RAT answers being studied (at the beginning (1), middle (2), or end (3) of RAT task).

### 2.2 Results and discussion

The data for the experiments are provided in the Open Science Framework at https://osf.io/2nvsg/. In all analyses reported below, we considered only those pairs that were correctly learned, according to our criterion test at the end of the study phase (see Methods); however, our results and all conclusions were qualitatively similar when all pairs were considered (For full reporting of criterion test performance and RAT data, not conditional on correct initial learning on the criterion test, please see Appendices B.1. and B.2, respectively). Complementary multi-level logistic regression models incorporating random slopes and intercepts for subject and item were also fit for each of the major analyses reported in-text, producing similar results and identical conclusions; for the sake of exposition, we report only the ANOVAs.

#### 2.2.1 Effects of retrieval suppression

**Fig. 2:**
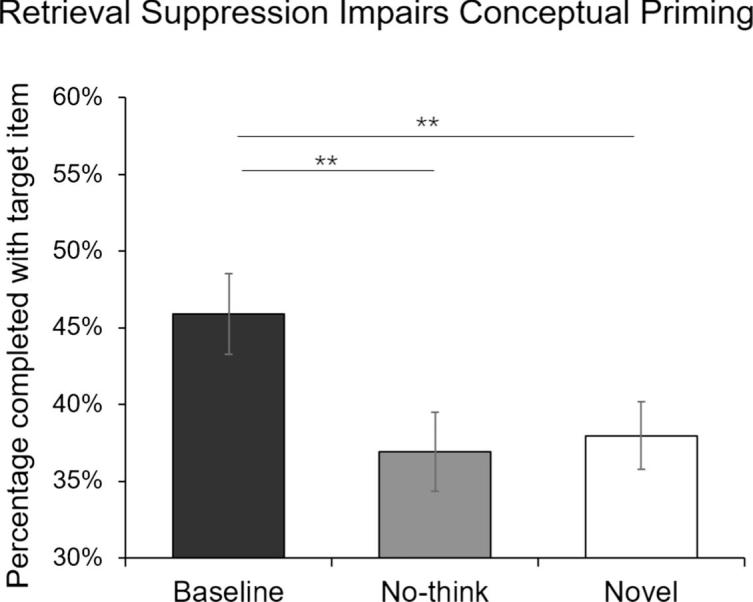
Mean percentage of RAT problems solved with the intended target item for the Baseline, No-think, and Novel conditions for Experiment 1. ^**^p < .01. Error bars represent ± 1 SEM.

Fig. 2 illustrates the percentage of correctly solved RAT problems for the No-think, Baseline and Novel conditions. A repeated-measures analysis of variance (ANOVA) showed a significant main effect of Suppression Status, *F*(1.90, 55.04) = 6.89, *p* = .003, η^2^ = 0.19, Greenhouse-Geiser correction. Paired t-tests revealed impaired performance in the No-Think condition when compared to the Baseline condition, *t*(29) = 3.16, *p* = .004, *BF*_10_ = 10.61, mirroring suppression-induced forgetting effects found in explicit (Anderson & Green, 2001) and perceptually-driven implicit (Kim & Yi, 2013) memory tests.^3^ Studying the solution words beforehand benefited participants’ performance in solving the RAT problems, as revealed by a significant conceptual priming effect for the Baseline condition when compared with the Novel condition, *t*(29) = 2.89, *p* = .007, *BF*_10_ = 5.94. However, evidence for a priming effect due to prior exposure was eliminated in the No-Think condition, *t*(29) = −0.45, *p* = .660., *BF*_01_ = 4.68. These findings indicate that items that are putatively inhibited during retrieval suppression trials are less likely to be later produced as solutions to a semantic association task.

#### 2.2.2 Effects of retrieval suppression in unaware participants

To assess contamination by explicit memory, we first examined participants’ reported awareness of relationships between the studied word pairs from the Think/No-Think phases of the experiment and responses provided during RAT problem solving. Out of 30 participants, only 6 reported noticing that some of the solution words had been studied previously (see Appendix C for post-experimental Questionnaire data). This low rate of awareness likely arose because (a) only 40% of the RAT problems had solutions that related to the earlier phases, (b) participants only solved, on average, 40% of those primed items (yielding 16% of the 60 trials, on average, with responses that could be recognized), and (c) the lack of relatedness of RAT clues to any cue words. Of the 6 participants who did report awareness, no participant reported explicitly retrieving items to assist in their problem solving.

To examine whether explicit retrieval produced the observed suppression-induced forgetting effect, we omitted the 6 participants reporting awareness and reconducted our analyses on the unaware group (*N* = 24). Our findings were largely unchanged. As in the overall sample, we observed a main effect of Suppression Status (Baseline versus No-Think versus Novel), *F*(1.88, 43.32) = 5.14, *p* = .011, η^2^ = 0.18 (Greenhouse-Geiser correction). Moreover, suppression-induced impairment was detected again in the RAT (Baseline vs. No-Think), *t*(23) = 2.86, *p* = .009, *BF*_10_ = 5.36. We observed a conceptual priming effect in the Baseline condition, *t*(23) = 2.10, *p* = .047, *BF*_10_ = 1.37, but not in the No-Think condition, *t*(23) = -1.18, *p* = .252, *BF*_10_ = 2.51, when compared against performance in the Novel condition. These findings indicate that retrieval suppression decreases the accessibility of the target words, impairing concept generation, suggesting that inhibition reduces accessibility of items at the conceptual level.

## 3 Experiment 2: Can the indirect influence of suppression be explained by explicit Retrieval?

Although the suppression effect observed in Experiment 1 appears to be implicit, one might be concerned with relying on participants’ post-experimental subjective reports to reach this conclusion. It is possible, for example, that people may not remember accurately whether they were aware of relationships between their responses and earlier studied items, during the RAT; alternatively, they may remember, but may not report awareness truthfully because they believe they were not supposed to notice—a demand characteristic. A complementary approach to addressing this issue would be to instruct participants directly to use explicit retrieval to solve RAT problems, so that the consequences of adopting this strategy can be ascertained. If suppression effects in Experiment 1 arose from contamination by explicit retrieval, then asking people to use explicit retrieval ought to increase the size of this effect by ensuring that the majority of the participants use episodic memory. Experiment 2 tested this hypothesis by contrasting two groups employing different strategies for solving RAT problems: the Implicit Instruction group, which was tested with the implicit procedure of Experiment 1, and the Explicit Instruction group, which was instructed to use explicit memory retrieval. Direct comparison of these two groups should reveal larger suppression effects in the Explicit group than in the Implicit group if explicit memory contamination caused the suppression effect in the Implicit group. To complement this manipulation, we retained the post-experimental questionnaire from Experiment 1, to see whether dividing Implicit Testing participants by self-reports of Explicit retrieval strategies yielded a pattern similar to that observed in our manipulation of test instructions.

Although it is necessary to consider the role of explicit retrieval strategies in producing the findings of Experiment 1, theoretical considerations suggest that explicit retrieval ought to reduce, not increase evidence for suppression-induced forgetting on our problem solving test. This possibility follows from the *masking hypothesis* of covert cuing (introduced in work on retrieval-induced forgetting; Anderson, 2003) adapted to the Think/No-Think task. By this hypothesis, recruiting episodic memory to solve RAT problems involves thinking back to the Think/No-Think phase to covertly recall studied targets that could suggest solutions. A studied target (e.g., TAPE) can either be recalled directly via its association to the context, or instead by first recalling the cue word with which that target was paired (e.g., NYLON, from NYLON-TAPE). Critically, the cue words from No-Think pairs (e.g., NYLON) should be far more accessible than those from Baseline pairs, owing to extensive repetition (10 times) during the TNT phase. If so, RAT problems for No-Think items should be easier to solve with explicit retrieval because people can more often recall the relevant cue word for the correct answer. Predictions of a similar masking hypothesis have been confirmed in research in retrieval-induced forgetting (Weller, Anderson, Gomez-Ariza, & Bajo, 2013).

As a secondary goal of this experiment, we tested key predictions of this covert masking hypothesis. This hypothesis predicts: (a) cue words from No-Think pairs should be more accessible than cues from Baseline pairs; (b) the greater the advantage in No-Think cue accessibility, the smaller the suppression effects should be; and (c) the foregoing prediction in (b) should arise only for the Explicit condition, in which participants are directed to use covert cuing, and not in the Implicit condition, in which (if truly implicit) participants would not use covert cuing. To test these predictions, we followed the RAT task with an additional “reverse” recall test. In this task, we gave participants each of the targets from earlier studied pairs and asked them to retrieve the cue word that went with it (e.g., If NYLON-TAPE had been a pair, they would be given TAPE and asked to recall the cue word). Prior work using this reverse recall test has compellingly demonstrated that No-Think cue words are substantially more accessible than are Baseline cues (Racsmany, Conway, Keresztes, & Krajcsi, 2012). We used this reverse recall advantage as an estimate of the likely boost in accessibility that No-Think cues enjoyed during the covert cuing process that took place during the earlier RAT task. If the reverse recall advantage successfully estimates this accessibility advantage and if the covert masking hypothesis is correct, Explicit instructions should reduce suppression-induced impairment, with the degree of reduction predicted by the reverse recall advantage.

### 3.1 Method

#### 3.1.1 Participants

Sixty right-handed native English speakers (35 female participants, mean age = 23.16 years, *SD* = 3.77) were recruited from the MRC Cognition and Brain Sciences Unit participant panel. The participants had no reported history of head injury, neurological disease, or learning disability and none were red/green color-blind. No participants took part in prior studies of retrieval suppression in the laboratory. The project was approved by the Cambridge Psychology Research Ethics Committee, and all participants gave written informed consent. The participants were randomly assigned to the Implicit and Explicit groups. The sample size was again determined based on convention for studies of retrieval suppression and item counterbalancing considerations.^4^

#### 3.1.2 Materials

The same materials were used as in Experiment 1.

#### 3.1.3 Design

Experiment 2 employed a 3 × 2 mixed design, with the Suppression Status of an item (Baseline, No-Think, Novel) and Test Instruction (Implicit, Explicit) manipulated within and between subjects, respectively. In the Implicit group, the TNT and RAT procedures of Experiment 1 were employed. In contrast, participants in the Explicit group underwent the same procedures except that they were told, immediately prior to the RAT, that some of the target words from the prior studied word pairs could be used as the solutions to the RAT problems that they were about to solve. This difference in test instruction was expected to amplify the involvement of explicit memory retrieval during the RAT, allowing us to determine how explicit strategies influenced the size of the SIF effect on that test.

In addition to the change in test instructions, we included an additional test after the RAT phase: a reverse association recall test. The reverse recall test was included to test whether cue words in the No-Think condition were significantly more accessible than cue words in the Baseline condition, due to TNT training, and whether this retrieval advantage predicted the size of the SIF effect on the RAT in the Explicit group.

#### 3.1.4 Procedure

Whereas the procedures of the Implicit group were the same as in Experiment 1, the Explicit group received explicit recall instructions prior to the RAT phase. The Explicit group was told that “Some of the RAT problems were solvable by target words from the studied word pairs”. In solving the RAT puzzles, participants were encouraged to “First try remembering a word that they studied to see if it fits as a solution, and, if nothing comes to mind, then simply solve the puzzle using their general knowledge and creative insight”.

After the RAT phase, the reverse recall task was administered. On each trial in this task, a solution word of one RAT problem appeared. For each solution word, participants were asked to retrieve the cue word that was associated with the solution word during the original study phase of the Think/No-Think task. All 60 RAT solutions were tested, even though only 24 items were targets from studied pairs. Participants were told to generate the original cue word only if they recognized a target word as having been studied. No time limit was given.

### 3.2 Results and discussion

Only pairs that participants learned were analyzed based on the results from the criterion test; however, all relevant results were significant based on inclusion of all pairs as well (See Appendix B for full results of unconditionalized analysis).

#### 3.2.1 Effects of retrieval suppression on RAT performance

The percentages of correctly solved RAT questions were submitted to a 3 × 2 mixed-design analysis of variance (ANOVA) with Suppression Status (Baseline vs. No-Think vs. Novel) as a within-subject factor and Test Instructions (Implicit vs. Explicit) as a between-subject factor. The main effects of Suppression Status, *F*(1.66, 96.25) = 14.20, *p* < .001, η_p_^2^ = 0.20 (Greenhouse-Geiser correction) and Test Instruction, *F*(1, 58) = 6.03, *p* = .017, η_p_^2^ = 0.09, were both significant. The interaction between these two factors was also significant, *F*(1.66, 96.25) = 4.35, *p* = .021, η_p_^2^ = 0.07 (Greenhouse-Geiser correction), indicating that the pattern of RAT performance in our Suppression Status conditions differed as a function of Test Instruction.

**Fig. 3:**
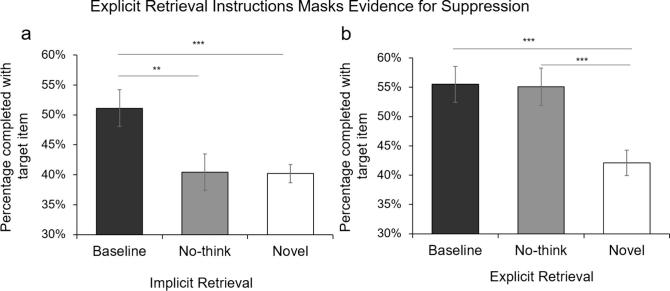
Mean percentage of RAT problems solved with the intended target item for the Baseline, No-think, and Novel conditions for Experiment 2, separately for the (a) Implicit- and (b) Explicit-retrieval groups. ^**^p < .01, ^***^p < .001. Error bars represent ± 1 SEM.

We investigated simple effects between each pair of conditions for the Implicit and Explicit instruction groups separately with paired t-tests. In the Implicit group, a significant suppression-induced impairment was detected, such that RAT problems were solved less often when their solutions had been voluntarily suppressed during earlier No-Think trials (Fig. 3a, Baseline versus No-Think: *t*(29) = 3.16, *p* = .004, *BF*_10_ = 10.61). A conceptual priming effect was detected for the Baseline condition when compared with the Novel condition (*t*(29) = 4.59, *p* < .001, *BF*_10_ = 320.60). This priming effect was abolished when items had been suppressed (No-Think versus Novel: *t*(29) = -0.09, *p* = .928, *BF*_01_ = 5.12), replicating the findings in Experiment 1.

Crucially, however, this pattern changed when participants were given explicit memory instructions to solve RAT problems (see Fig. 3b). No evidence of suppression-induced impairment was found in the No-Think condition when compared with the Baseline condition (*t*(29) = 0.10, *p* = .925, *BF*_01_ = 5.12). Indeed, with explicit memory instructions, superior RAT performance was found both in the Baseline condition, *t*(29) = 4.52, *p* < .001, *BF*_10_ = 269.10, and in the No-Think condition (*t*(29) = 4.10, *p* < .001, *BF*_10_ = 95.29), relative to performance in the Novel condition. These findings suggest that the suppression-induced impairment observed in the Implicit group is unlikely to reflect contamination by explicit memory. Rather, the involvement of explicit retrieval in the task reduces evidence of suppression-induced impairment, consistent with the covert masking hypothesis. We turn to this hypothesis next.

#### 3.2.2 Testing the masking hypothesis with reverse recall performance

**Fig. 4:**
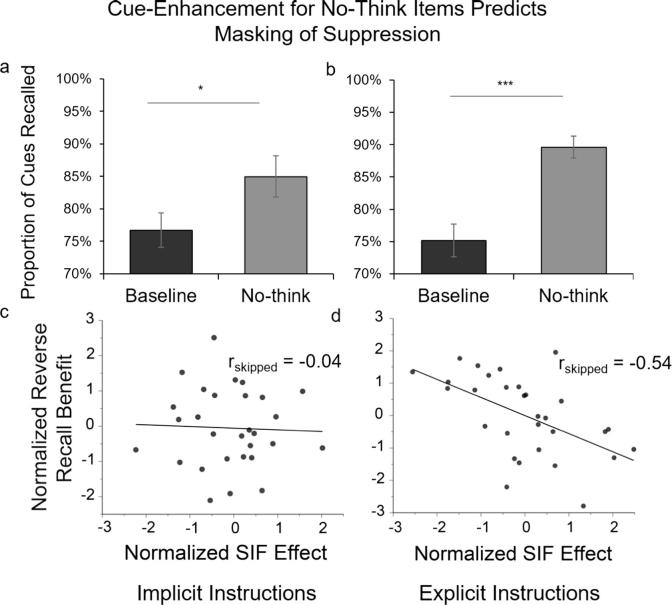
Reverse recall performance reveals cue-enhancement for No-Think cues, for both the Implicit (a) and Explicit conditions (b). ^**^p < .01, ^***^p < .001. Error bars represent ± 1 SEM. (c) The correlation between suppression-induced impairment and cue-enhancement in the Implicit group was not significant (r-skipped = −0.04, CI_95%_ = [−0.34, 0.26]), but it was in the Explicit group, as predicted by the covert masking hypothesis (d) (r-skipped = −0.54, CI_95%_ = [−0.74, 0.29]).

The absence of suppression effects in the Explicit Instruction condition is consistent with our covert masking hypothesis. To test specific predictions of this hypothesis, we measured the degree to which repeatedly viewing the cue words for No-Think items during the TNT phase had enhanced their accessibility in memory. To quantify this, we measured participants’ ability to recall cue words upon seeing the target words for each condition (i.e., “reverse recall”). A 2 (Suppression Status: No-Think vs. Baseline) by 2 (Instruction group: Implicit vs. Explicit) mixed-design ANOVA was performed with Instruction Group as a between-subject factor. We observed a main effect of condition which showed reverse recall benefits for cues to No-Think target words, relative to Baseline target words *F*(1.00, 58.00) = 28.60, *p* < .001, η_p_^2^ = 0.33 (Greenhouse-Geiser correction). The main effect of group, *F*(1, 58) = 0.28, *p* = .598, η_p_^2^ = 0.01, and the interaction effect between the two factors, *F*(1.00, 58.00) = 2.14, *p* = .149, η_p_^2^ = 0.04 (Greenhouse-Geiser correction) were not significant (see Fig. 4a and b). The reverse recall benefit was found in both the Implicit (No-Think vs. Baseline: *t*(29) = 2.75, *p* = .010, *BF*_10_ = 4.45) and Explicit groups (No-Think vs. Baseline: *t*(29) = 4.81, *p* < .001, *BF*_10_ = 557.50). The reverse recall benefit supports the prediction that the cue words would be more episodically accessible for No-Think items than for Baseline items.

To test the additional key predictions of the covert masking hypothesis, we correlated suppression-induced impairment on the RAT with the reverse recall benefits observed for the No-Think cues, separately in the Implicit and Explicit groups. Both suppression-induced impairment (Baseline – No-Think) and the reverse recall benefit for No-Think cue words (No-Think - Baseline) were *z-*normalized within each item counterbalancing condition, as in prior work (see, e.g. Anderson et al., 2004, Hulbert and Anderson, 2018). According to the masking hypothesis, only people given Explicit recall instructions would have sought to recall previously studied items during the RAT task and therefore would have retrieved the cue words to help in generating answers for the RAT problems. If so, and if the reverse recall advantage for No-Think cues estimates the advantage subjects would have enjoyed in recalling those No-Think cue words during the RAT, then the larger this advantage, the smaller the suppression effects should be for the Explicit group. Consistent with this masking hypothesis, suppression-induced impairment on the RAT test was negatively correlated with the reverse recall benefits in the Explicit group (Fig. 4d, r_skipped_ = −0.54, [−0.74, −0.29] bootstrapped 95% CI). That is, people who showed a greater advantage for No-Think cue word recall (compared to Baseline cue recall) also showed smaller suppression-induced impairment on the RAT. We observed no significant relationship, however, between suppression-induced impairment and the reverse recall benefit in the Implicit group (Fig. 4c, r_skipped_ = −0.04, [−0.34, 0.26] bootstrapped 95% CI). This latter finding verified that (1) RAT problem solving accuracy in the Implicit group was not affected by the greater accessibility of the cue words for the RAT solutions, and (2) the reduced suppression-induced impairment in the Explicit group was likely due to masking effects arising from covert retrieval of the cue words during RAT problem solving.

#### 3.2.3 Combined analysis of implicit remote associates test

**Fig. 5:**
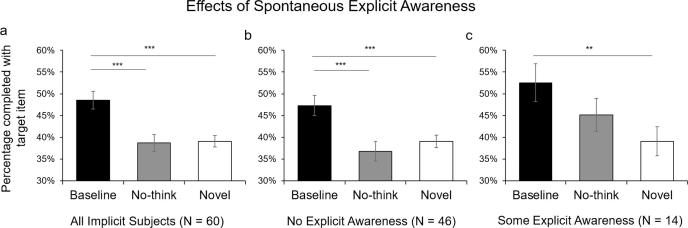
Mean percentage of RAT problems solved with the intended target item for the Baseline, No-think, and Novel conditions for the aggregate analysis of Implicit Retrieval groups across Experiments 1 and 2. Overall, suppression reduced conceptual implicit memory (a). Participants who reported no awareness of solutions being studied in the experiment showed robust suppression (b), whereas those reporting explicit awareness of the link to the earlier study phase shows less persuasive suppression (c). ^**^p < .01, ^***^p < .001. Error bars represent ± 1 SEM.

Experiments 1 and 2 suggest that suppressing episodic retrieval reduces the indirect influence of suppressed content on later thinking. The manipulation comparing the Implicit with Explicit test instructions in Experiment 2 confirms that the suppression-induced inhibition observed under Implicit Instructions was unlikely to be caused by contamination by explicit memory retrieval. To further test this possibility, we examined the relationship between post-experimental reports of Explicit Strategy use in the Implicit condition and the size of the suppression-induced impairment effect. To do this, we focused our analyses on only those participants who reported no awareness of the connections between the studied items and the RAT solutions, combining participants from both Experiments 1 and 2 for greater power. Forty-six participants reported that they were unaware that the solution words were sometimes the same as ones they studied. A repeated measures ANOVA revealed a significant main effect of condition in the Unaware group (Fig. 5b, F(1.88, 82.73) = 12.26, *p* < .001, η^2^ = 0.22, Greenhouse-Geiser correction). Inspections on the simple effects showed a significant suppression-induced impairment effect on RAT problem solving (Baseline vs. No-Think: *t*(45) = 4.22, *p* < .001, *BF*_10_ = 203.50). Moreover, whereas a significant priming effect was detected for Baseline items (Baseline vs. Novel: *t*(45) = 3.91, *p* < .001, *BF*_10_ = 84.29), no difference was found between the No-Think and Novel conditions (*t*(45) = -1.10, *p* = .279, *BF*_01_ = 3.55), indicating that priming had largely been eliminated.

A different pattern was found for people who noticed the connection between the studied word pairs and the RAT problems at some point during the test. A significant main effect of condition was observed (Fig. 5c, F(1.67, 21.67) = 5.88, *p* = .012, η^2^ = 0.31, Greenhouse-Geiser correction). Participants did not show reliable suppression-induced impairment on the RAT (Baseline vs. No-Think: *t*(13) = 1.58, *p* = .139, *BF*_01_ = 1.35), although the conceptual priming effect in the No-Think condition was not significant either (No-think vs. Novel: *t*(13) = 1.74, *p* = .105, *BF*_10_ = 1.11). In contrast, the conceptual priming effect was significant for Baseline items (Baseline vs. Novel: *t*(13) = 3.86, *p* = .002, *BF*_10_ = 22.00). These combined analyses further support that the suppression-induced impairment in RAT performance truly reflects the extension of suppression to novel thought processes without awareness of the participant, and that explicit memory contamination is an unlikely cause.

The combined analysis of Experiments 1 and 2 highlights a remarkable feature of the current findings that bears strong emphasis: Retrieval suppression did not merely reduce conceptual priming, it seemingly abolished it altogether (with Bayes Factors providing substantial support for the null hypothesis). Thus, participants behaved as though they had never seen the No-Think items before, showing no measurable benefit of prior exposure. Indeed, in unaware participants, no-think items were produced at a rate numerically lower than novel items. These findings provide compelling evidence against unconscious persistence at the conceptual level, suggesting that a key premise of classical ideas about the indirect effects of suppression may be incorrect. In these studies at least, suppression was, in fact a highly effective means of diminishing the impact of suppressed contents on ongoing cognition.

## 4 General discussion

The idea that suppressed thoughts persist in the unconscious, indirectly influencing our thinking outside of our awareness, is nearly as old as psychology itself. In this article, we tested a simple premise of this proposal: that the semantic content of a suppressed memory implicitly survives efforts to suppress it and, unthwarted, continues to promote thoughts involving that content in ostensibly unrelated contexts. If so, then in a thinking task in which conceptual priming arises, prior suppression of a memory should have little sway over the priming it shows. To test this, we replaced the episodic recall test normally used to measure suppression-induced forgetting with a test that indirectly measured the activation of semantic representations underlying the suppressed content: the remote associates test. We presented participants with what we characterised as “creativity problems” that tested their ability to discover the hidden concept linking three novel cue words. Although the large majority of these problems were unrelated to the prior TNT task, on critical trials, the three cues were semantic associates to a previous target item (but unrelated to that target’s studied cue word). In the Implicit testing condition, we made no mention of connections to the TNT task, allowing prior exposures of targets to influence problem solving indirectly. Results from two experiments showed that suppressing No-Think words affected conceptual priming; it reduced the likelihood of No-Think items being provided as solutions, relative to when those items were studied, but not suppressed (i.e., Baseline items). Strikingly, retrieval suppression abolished, for No-Think items, the priming benefits enjoyed by studied items. Essentially, suppression was so effective that participants behaved as though they had not seen the No-think items.

We considered whether participants noticed the relationship between the RAT and the TNT task. If so, the RAT may not have measured conceptual priming, and SIF might instead reflect suppression of explicit memory. Several observations make this unlikely, however. First, participants reported the RAT to be enjoyable and challenging, and so their attention was focused on how the three, apparently unrelated words, might be related. The problems were all solvable using general knowledge and we placed no constraints on their answers, apart from the solution “making sense”. Second, we disguised the relationship between the tasks in multiple ways, including (a) using cues in RAT problems that were unrelated to the original cue words to the solution from the TNT task, (b) ensuring that few of the intended RAT solutions could be linked to studied items (maximum of 24 out of 60, or 40%; in actual practice, given the solution rate of 40–50%, this meant that only 16−20% of the items produced by participants matched those studied), and (c) using a cover story to characterize the two tasks as different experiments. These procedural controls worked well: less than 30% of the participants reported that some of the solutions they generated had been studied. When we restricted our analysis to the 70% unaware participants, suppression effects were, if anything, stronger than in the overall sample. Third, we probed what would happen if participants had explicitly retrieved items, by asking them to recall studied items to solve RAT problems in the Explicit group of Experiment 2. Contrary to the explicit contamination hypothesis, participants instructed to retrieve studied items showed no suppression-induced impairment. Indeed, we found reduced SIF in the Implicit condition for participants reporting awareness of having studied some of the targets (Fig. 5c) when compared with the unaware group (Fig. 5b). Together, these results provide clean evidence that decreased RAT solution rates for suppressed items do not arise from explicit contamination; rather retrieval suppression disrupts the indirect influence of suppressed content on an apparently unrelated thinking test.

The finding that explicit memory instructions in Experiment 2 eliminates suppression effects on the RAT is consistent with our extension of the masking hypothesis (Weller et al., 2013) to suppression-induced forgetting. By this hypothesis, when participants use episodic memory to retrieve responses for extra-list cues, they think back to the Think/No-Think task to recall studied material that could suggest solutions. Because cue words from No-Think pairs should be more accessible than those from Baseline pairs (from the massive repetition of No-Think cues in the TNT phase), participants should generate more cue words for No-Think RAT problems. RAT problems for No-Think items therefore should be more often solved using a cue composed of the remote associates and a covertly retrieved cue word, providing a compound cuing advantage large enough to mask inhibition of the No-Think target. To test this account, we followed the RAT with a task that asked participants to recall the cue words of the paired associates for the No-Think and Baseline targets. As in past work (Racsmany et al., 2012), participants recalled more No-Think than Baseline cue words. Critically, however, we found that the size of the recall advantage for No-Think over Baseline cues predicted suppression-induced forgetting in the Explicit condition: the larger the advantage, the smaller was suppression-induced forgetting. Thus, the suppression effect on explicit memory tests that use independent probes can be masked by covert cuing, as observed in retrieval-induced forgetting (Weller et al., 2013). Importantly, although we also found a No-Think cue recall advantage in the Implicit group, it did not predict the suppression effect on the RAT. This indicates that cue accessibility advantage for No-Think items only matters when participants use an explicit memory strategy, consistent with our extension of the masking hypothesis.

### 4.1 Relation to other findings

The current findings do not stand alone in suggesting that suppression alters the accessibility of the semantic content underlying a suppressed thought (Hertel et al., 2012, Hertel et al., 2018, Taubenfeld et al., 2018). Our findings, however, establish more diagnostic evidence that inhibition produces the phenomenon and compellingly demonstrate an indirect influence. For example, unlike Hertel et al. (2012) free association test, our remote associates test uses cues that are unassociated to the studied cues for their solutions. Particularly, RAT creativity problems could only be solved by generating correct associations shared by three independent cues with one common word. In this context, focusing on any one cue would not be sufficient to generate the correct solution and would instead cause fixation and slow down the process (Gomez-Ariza et al., 2017, Koppel and Storm, 2014, Storm and Angello, 2010). Given these features, our RAT task constituted a particularly good independent probe test (Anderson and Green, 2001, Anderson and Spellman, 1995) unlikely to be affected by interference (see, e.g., Wang et al., 2015), allowing a stronger inference of inhibition. The un-relatedness of the RAT cues to the cues also disguised the relation between our final test and the TNT task; together with the low proportion of trials completable by studied items, our procedure permits confidence that performance reflects the unconscious influence of study events, unlike in the foregoing studies. The finding of suppression-induced forgetting on the current test, however, suggests that these other observations may truly reflect, in part, the effects of inhibition.

The null suppression effect in the Explicit group of Experiment 2 has important implications for measuring inhibition with independent cues. This finding provides converging evidence for the masking hypothesis of covert cuing (Anderson, 2003, Weller et al., 2013), extending it suppression-induced forgetting. Our correlational analysis relating the size of the accessibility advantage for No-Think cue words to reductions in suppression-induced forgetting confirms key predictions of this hypothesis and shows that masking effects are a concern when Explicit testing is used. Given this finding, one might wonder why suppression-induced forgetting has been observed on independent probe tasks in past studies, given that all such tasks also have employed extra-list cuing and explicit instructions (Anderson & Green, 2001). One mitigating factor, however, is that the standard independent probe task involves an extra-list category with an item specific cue (a letter, e.g. for Roach, Insect-R___). Item-specific cues may reduce reliance on covert free recall, leading participants to generate answers from semantic knowledge, and test them against episodic memory (Anderson, 2003); if so, covert masking may be irrelevant to this process, which circumvents the cue word.

Although we have focused on how suppressing an unwanted memory affects its semantic influences, suppression also reduces the indirect expression of non-semantic aspects of experience. For example, suppressing retrieval of visual images makes it harder to perceive the objects in those images when they are later encountered (Gagnepain et al., 2017, Kim and Yi, 2013); and when the images are aversive scenes, it reduces the negative valence perceived in them (Gagnepain et al., 2017). One interesting exception, however, is that suppression may not affect orthographic priming (Angello, Storm, & Smith, 2015). Angello et al. (2015) built on orthographic blocking effects, in which incidental encoding (e.g. of ANALOGY) makes it harder to solve word-fragment completion problems for orthographically similar words (e.g. when trying to solve A_L_ _GY, participants were less likely to generate ALLERGY because they had encoded ANALOGY). Angello et al. (2015) tested whether suppressing the “blocker” (ANALOGY) prior to word-fragment completion reduced the blocking effect. It did not. They did, however, observe reduced blocking when they gave explicit memory instructions on the word-fragment completion task. This pattern--the opposite to that observed here, led Angello et al. (2015) to argue that their suppression effect disrupted episodic traces. More generally, these findings suggest that suppression may not affect orthographic representations.^2^

Retrieval suppression contrasts with other motivated forgetting procedures such as directed forgetting in whether it affects implicit memory. The directed forgetting procedure asks people to intentionally forget memory items, via one of two methods: the item-method (e.g., Bjork and Woodward, 1973, Fawcett et al., 2016; see Anderson & Hanslmayr, 2014, for a review) or the list-method (e.g., Bjork, 1989, Geiselman et al., 1983; see Anderson & Hanslmayr, 2014, and Sahakyan, Delaney, Foster, & Abushanab, 2013, for reviews). In the list-method, participants study two lists, with either a forget or remember instruction given after the first list; after the second list, a brief distracting task follows and then a recall test. On this test, people recall the first list more poorly when it is followed by a forget, instruction. Importantly, these forgotten items show repetition priming on indirect tests (Basden, 1996, Basden et al., 1993, Bjork and Bjork, 1996, Bjork and Bjork, 2003, Paller, 1990), in contrast with the present findings. One reconciliation of these contrasting results is that list-method directed forgetting impairs recall not by inhibiting individual items, but instead by making people forget the “mental context” of the first list (for supportive neuroimaging and behavioral evidence, see Manning et al., 2016, Bauml et al., 2010, Sahakyan and Kelley, 2002; however, see evidence for selective directed forgetting that questions the sufficiency of this view; Aguirre et al., 2014, Aguirre et al., 2017, Delaney et al., 2009, Kliegl et al., 2013). This context-forgetting process may involve inhibiting contextual representations (e.g., Anderson, 2005, Anderson and Hanslmayr, 2014, Bauml et al., 2008, Bauml et al., 2010, Hanslmayr et al., 2012; or a contrasting view, see Sahakyan et al., 2013, and Sahakyan & Kelley, 2002). Because recalling first-list items requires access to the list-1 context, people fail to retrieve studied items. Critically, by this view, because implicit tests do not require access to the study context, performance should remain intact, as is found. In contrast, forgetting procedures that target individual memories, either via retrieval-suppression (e.g., the Think/No-Think task) or by encoding disruption (e.g. item-method directed forgetting), or retrieval-induced forgetting disrupt the unwanted trace itself (Gomez-Ariza et al., 2017, Valle et al., 2019) leading to forgetting on indirect tests (see, e.g., Basden et al., 1993). These diverging findings point to the importance of restricting our conclusions to retrieval suppression of intrusive memories.

Retrieval suppression also differs from another procedure used to study thought suppression: the White Bear paradigm (Wegner, Schneider, Carter, & White, 1987). This paradigm instructs participants to not think of a specific thought (e.g. a white bear) for 5-minutes, and, during this time, to report when it comes to mind. In this method, participants have difficulty avoiding the unwanted thought, a finding taken to show the inefficacy of thought suppression (Wegner, 1994). Intriguingly, suppressing a thought under stress, time pressure or divided attention, increases the likelihood that it will influence behavior indirectly (Wegner & Erber, 1992; see Wegner, 2009, for a review). For example, under time pressure, people are more likely to free associate the word “house” given the words home, door, brick or roof, if they have been asked to suppress “house” for the past five minutes and continue to suppress during free association. Based on these findings, Wegner and Smart (1997) proposed that suppressed thoughts, though not in awareness, remain hyper-accessible, in a state of “deep semantic activation”, paralleling classical notions of persisting unconscious influences. Critically, however, the White Bear task makes suppression unlikely to succeed. In making explicit reference to a particular forbidden thought, suppression becomes impossible, because simply remembering the task’s stated purpose necessarily violates of the task goal: the forbidden thought is integrated with the task set, ensuring that it is intermittently refreshed. It is thus unsurprising that, if distracted, people free associate House during a task that requires periodic thoughts about House. This contrasts with retrieval suppression, which does not mention the thought to be avoided; rather, participants simply are asked to suppress awareness of memories given reminders. This difference in *goal-integration* may account for discrepancies in suppression success between retrieval suppression and the White Bear paradigm (Anderson and Huddleston, 2012, Engen and Anderson, 2018). If so, the White Bear task may misrepresent the true utility of suppression and may not measure processes critical to controlling intrusions. Consistent with this, a meta-analysis (Magee, Harden, & Teachman, 2012) of 33 White Bear studies found no differences in suppression success or in rebound effects across patients suffering from intrusive symptomatology and controls. If this task is insensitive to clinical deficits in suppression, the lessons it holds about how suppression affects unconscious influences may not be instructive.

We suggest that retrieval suppression provides a particularly useful cognitive model of how people control unwanted memories and thoughts in daily life—a procedure with advantages over the White Bear and directed forgetting tasks as a model of motivated forgetting. Directed forgetting effects are only observed for an immediately preceding time period, making their relevance to clinical concerns more constrained and indirect. Moreover, directed forgetting involves inhibitory control, but it does not address a situation clearly relevant to combating intrusive thoughts: confronting unwelcome reminders and needing to prevent awareness of the offending content. Such reminders can occur at any time and may induce intrusions of events or thoughts that happened today, yesterday, or 10 years ago; indeed, the thoughts may be of future fears and may not have ever, in fact occurred (Benoit et al., 2016). Studying retrieval suppression permits the study of memory control in these situations, which are relevant to understanding key symptoms of psychological disorders. Given these considerations, the fact that suppressing episodic retrieval reduces the indirect effects of a suppressed experience on conceptual, affective, and perceptual measures is highly germane to any indirect influences this behaviour may have on mental health. This recurring pattern across indirect tests, moreover, calls out for an account of why explicit and implicit memory are both affected by suppression, despite neuropsychological dissociations of these forms of memory.

### 4.2 A Framework for understanding the impact of suppression on indirect tests

How does suppressing retrieval of an episodic memory also impair implicit memory? Such a dependency seems at odds with work on multiple memory systems, which emphasizes dissociations between explicit and implicit memory. Indeed, the classical memory systems view emphasizes that these forms of memory are independent, with the former supported by the medial temporal lobes and the latter by distinct cortical and subcortical systems (Gabrieli, 1998, Schacter et al., 1993, Squire, 1992, Tulving and Schacter, 1990). Supporting this possibility, amnesic patients can lack conscious memory for an event yet reveal its unconscious influences through intact emotional conditioning (Bechara et al., 1995), repetition priming (Hamann and Squire, 1997, Schacter et al., 1993, Schacter, 1987, Tulving and Schacter, 1990), and other forms of implicit memory (Gabrieli, 1998, Squire, 1992). Even healthy participants can show priming of visual objects more than a decade after exposure, with no explicit memory of the items (e.g., Mitchell et al., 2018). Moreover, work on suppression-induced forgetting emphasizes the role of prefrontal control processes that down-regulate hippocampal activity to disrupt retention of the unwanted memory (Anderson et al., 2004, Anderson et al., 2016, Benoit and Anderson, 2012, Benoit et al., 2015, Depue et al., 2007, Gagnepain et al., 2014, Gagnepain et al., 2017, Hulbert et al., 2016, Levy and Anderson, 2012, Liu et al., 2016, Schmitz et al., 2017). Given these considerations, one might have expected suppression to preserve implicit memory, in line with the idea that suppressed contents continue to wield unconscious influence. Indeed, we previously endorsed this possibility, which the current work shows is incorrect (Anderson, 2006).

**Fig. 6:**
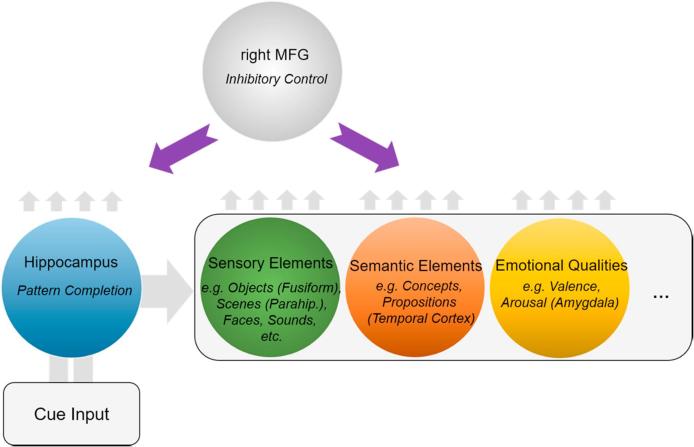
Schematic of the reinstatement dynamics predicted to bring about parallel inhibition of episodic memories and implicit influences (e.g., sensory, semantic, emotional) during memory intrusions. Cue input flows into the hippocampus (lower left), driving pattern completion (blue circle). Inhibitory control processes mediated by the right MFG target the hippocampus to suppress retrieval; if inhibition is too slow, pattern completion succeeds, sending re-entrant signals out of the hippocampus (large grey arrow) to reactivate traces in sensory, semantic, and emotion regions (colored circles). The particular cortical and sub-cortical regions reactivated depend on the specific event and the content represented. Intrusion-related reactivation is predicted to trigger parallel inhibition of these structures and the hippocampus by the right MFG (downward arrows), via polysynaptic pathways yet to be fully understood. (For interpretation of the references to colour in this figure legend, the reader is referred to the web version of this article.)

Despite strong dissociations between explicit and implicit memory in neurological patients, in healthy brains, the hippocampus interacts with neocortical areas to support intentional retrieval and also implicit memory (Hannula and Ranganath, 2009, Sheldon and Moscovitch, 2010, Wimmer and Shohamy, 2012). During voluntary retrieval, perceptual reminders initiate inputs to the hippocampus that are believed to elicit pattern completion, which, via reentrant connectivity, can reinstate sensory neocortical patterns that contributed to the initial experience (Danker and Anderson, 2010, McClelland et al., 1995). With involuntary retrieval, a similar interaction may arise, supported by a rapid process (Hannula and Greene, 2012, Moscovitch, 2008) in which cue input automatically reinstates associated content in neocortex. Based on these dynamics, we proposed that during suppression, inhibitory control responds by targeting both hippocampal activity and reinstated content-specific representations (see Fig. 6; Gagnepain et al., 2014, Gagnepain et al., 2017, Hu et al., 2017). Thus, upon confronting an unwelcome reminder, if inhibitory control is not deployed quickly enough to stop pattern completion, the hippocampus may reactivate neocortical regions via re-entrant pathways. This reinstatement, experienced as an intrusion, triggers upregulation and retargeting of control at the hippocampus in parallel with regions in which reinstatement has occurred. For example, when the intruding memory is of a visual object, rapid reactivation in, and suppression of regions involved in conscious object perception should arise (Gagnepain et al., 2014); if a visual scene intrudes, reactivation in and suppression of parahippocampal place area activity should occur (Benoit et al., 2015, Gagnepain et al., 2017); and if the intrusion elicits negative affect, amygdala activity should be suppressed (Depue et al., 2007, Gagnepain et al., 2017). Targeting of non-hippocampal regions has important consequences: If the neocortical or subcortical traces reinstated during intrusions support implicit memory, then suppressing awareness of intrusions should disrupt unconscious expressions of the intruding content. The particular expressions disrupted should be dictated by the content reinstated, a proposal that we refer to as the reinstatement principle (Gagnepain et al., 2017, Hu et al., 2017).

If the reinstatement principle is correct, then the current findings suggest that the semantic content of suppression targets was reinstated when participants experienced intrusions. If so, then retrieval suppression should not only reduce activity in hippocampus, but also in regions representing semantic content, including the left temporal cortex. Moreover, temporal cortical suppression should be more pronounced during intrusions, mirroring intrusion-related down-regulations in the parahippocampal place area and the amygdala when people suppress aversive scenes (Gagnepain et al., 2017). Future work should test this possibility to understand the neural basis of semantic suppression and to confirm the generality of the reinstatement principle. More broadly, this view predicts suppressing unwanted memories or thoughts should limit core ideas underlying the suppressed content from re-emerging indirectly in later thoughts.

### 4.3 Implications

If suppression disrupts implicit memory, historical assumptions about persisting unconscious influences of suppressed content may have been incorrect. Given that suppression disrupts the implicit expression of intruding content, whether perceptual (Gagnepain et al., 2014, Kim and Yi, 2013), affective (Gagnepain et al., 2017) or semantic in nature, and given the neural machinery underlying these effects, how successfully forgotten experiences could continue to influence mental health--if they in fact do---remains to be clarified. It bears emphasis, however, that our findings do not question the existence of unconscious influences per se, an idea which is amply supported by implicit memory (Gabrieli, 1998, Schacter et al., 1993, Squire, 1992, Tulving and Schacter, 1990). Rather, the specific proposal that suppression preserves unconscious expressions of suppressed content is called into question.

Might unconscious expressions sometimes survive efforts to control unwanted thoughts? Several possibilities are compatible with our findings. First, other forms of experiential avoidance may not disrupt either explicit or implicit memory. For example, one can limit awareness of unwanted thoughts by avoiding reminders (Engen & Anderson, 2018). By this learned avoidance hypothesis, people adopt mental and physical habits that divert their attention from reminders, occupying awareness with neutral content. Avoiding reminders should pre-empt any need for inhibitory control to suppress retrieval, ensuring the content is not inhibited. Second, the simple denial of a feeling, a thought, or a memory, absent any attempt to suppress awareness, would not necessarily induce forgetting or impair implicit expressions. Third, implicit influences of an event can remain intact after it is episodically forgotten, provided that forgetting is accomplished passively, by the passage of time (Mitchell et al., 2018) or changes in mental context, even those induced by control (Basden et al., 1993, Bjork and Bjork, 1996). Finally, some types of psychological material might re-emerge after suppressing retrieval. For example, thoughts about psychological conflict (e.g. between desires and beliefs) might, after suppression, re-emerge precisely because the circumstances that create the mental conflict recur, reviving the suppressed content. By this view, the classical notion of persisting unconscious content may be accurate if restricted to certain categories of material.

What seems unlikely given the current and related findings, is the broad proposal that suppressing intrusive thoughts generally preserves unconscious expressions of memory. Given that intrusive memories and thoughts pervade psychiatric disorders and are often be driven by reminders, this conclusion carries noteworthy implications for common beliefs about the desirability of suppression as a coping process. Specifically, absent a cognitive control deficit that compromises suppression, suppression may effectively mitigate unwanted thoughts; at the very least, concerns based on indirect influences do not have strong empirical grounding. Before accepting this conclusion, however, more work must establish the generality of these suppression effects on implicit retention using complex materials (see, however, Hu, Bergstrom, Bodenhausen, & Rosenfeld, 2015, for an example), and over longer time scales. Although these issues must remain unaddressed here, our findings converge with recent demonstrations of implicit suppression effects in cautioning that a careful empirical re-examination of this long-standing idea is warranted.

## 5 Concluding remarks

The idea that suppressed thoughts and memories haunt us indirectly in later mental processes and in our behaviour spans the history of psychology and is a compelling narrative. Although cognitive psychology and cognitive neuroscience provide clear evidence for indirect influences of past experience in behavior, surprisingly little work tests whether such influences are immune to voluntarily forgetting, particularly to retrieval suppression. Does suppressing intrusive thoughts and memories, even if successful, leave remnants of experience in implicit memory that discreetly and perniciously influence mental life outside of our awareness? To our surprise, and contrary to our own previous conjectures about the lingering influences of suppressed traces (Anderson and Huddleston, 2012, Anderson and Levy, 2006, Anderson, 2005, Levy and Anderson, 2008), the current study and others reported recently (Gagnepain et al., 2014, Gagnepain et al., 2017, Hertel et al., 2012, Hertel et al., 2018, Hu et al., 2015, Kim and Yi, 2013, Taubenfeld et al., 2018) suggest that this view is incorrect.

The present research indicates that episodic retrieval suppression inhibits the semantic content underlying an episodic trace. We found diminished accessibility of suppressed content measured on a task that participants view as unrelated to the original suppression context; that shares no cues with the study episode; that prompts little awareness of the episodic memory; and that clearly could benefit from prior exposure. Strikingly, retrieval suppression fully eliminated the indirect benefits of prior exposure, as far as can be measured on our test. That we measured such effects with independent cues implies a general reduction in semantic accessibility consistent with inhibitory control (Anderson and Green, 2001, Anderson and Spellman, 1995, Hertel et al., 2012), and indicates a broader impact of episodic suppression than we had entertained initially. This pattern suggests that classical ideas about unconscious persistence of suppressed thoughts, which have affected modern clinical thinking about the utility of suppression, merit reconsideration. It is, after all, only natural, when worries about the future or sadness about the past seize us, to seek to remove those aversive thoughts from awareness and regain footing in our mental landscape; towards this end, retrieval suppression may be a fundamental mechanism in the armamentarium of emotion regulation (Engen & Anderson, 2018) for restoring our emotional balance, enabling us to move on with more productive goals and, ultimately, for allowing our sleeping dogs to lie.

## Declaration of interest

The authors declare no competing financial interests.

## Figures and Tables

**Table:** 

Cue word	RAT cues	Target word
LEVEL	Question/check/birth	MARK
VASE	Cotton/bathtub/tonic	GIN
MOSS	Chocolate/youth/soda	FOUNTAIN
SCREW	Sharp/tick/tie	TACK
GIANT	Flag/vault/totem	POLE
LIVER	Hot/butterflies/pump	STOMACH
LUXURY	Surprise/wrap/care	GIFT
ADVICE	Lecture/teach/piano	LESSON
MISERY	Officer/cash/larceny	PETTY
CUSTOMS	Man/order/air	MAIL
THIMBLE	Master/toss/finger	RING
SURFACE	Plush/floor/tiles	CARPET
PINE	Date/oil/oasis	PALM
LOOP	Rain/base/trip	ACID
SLAB	Roll/marmelade/fish	JELLY
BELT	Shoulder/ mayor/badge	SASH
NYLON	Measure/worm/video	TAPE
SCENE	Eye/defense/testify	WITNESS
POCKET	Sleeping/tea/trash	BAG
PLAQUE	Advert /standard/slogan	BANNER
SYSTEM	Computer/cable/social	NETWORK
PARTNER	Upper/service/gloss	LIP
GLACIER	Spill /tear /bucket	DROP
CALORIE	Dew/comb/bees	HONEY
SIDE	Board/blade/back	SWITCH
PUFF	Champagne/gum/burst	BUBBLE
HEEL	Ruby/ballet/bunny	SLIPPERS
PLAID	Superman/hood/cloak	CAPE
CHILL	Hay/virus/yellow	FEVER
FABLE	Myth/tale/urban	LEGEND
MOMENT	Jump/kill/bliss	JOY
FARMER	Trap/polar/claw	BEAR
SALOON	Rocking/wheel/cushion	CHAIR
INSIGHT	Essence/halo/mystical	AURA
CAPTION	Playing/credit/report	CARD
COLONEL	Life/color/coast	GUARD
HERB	Rag/milk/out	WEED
BIBLE	Proverb/hymn/chapel	PSALM
LIST	Right/cat/carbon	COPY
TIDE	Show /pier /row	BOAT
TOOLS	Sage/paint/hair	BRUSH
QUILL	Type/ghost/screen	WRITER
VOLUME	Gas /diving /fill	TANK
WALNUT	Bomb/cocoon/turtle	SHELL
BANKER	Tax/real/fourth	ESTATE
VINEGAR	Tuna/lettuce/bowl	SALAD
DILEMMA	Genius/scan/child	BRAIN
ANTIQUE	Lit/birthday/romantic	CANDLE
SIZE	French/car/fog	HORN
CENT	Coin/quick/spoon	SILVER
ROOT	Skunk/coleslaw/boiled	CABBAGE
PRANK	Humour/half /smart	WIT
CHORE	Opera/hand/dish	SOAP
BRUISE	Bump/throat/sum	LUMP
REGION	Line/patrol/town	BORDER
TUNNEL	Ford/gap/vehicle	BRIDGE
ENERGY	Iron/shovel/engine	STEAM
EMERALD	Poison/league/vine	IVY
MISSILE	Shine/beam/struck	MOON
TRIBUTE	Heaven/reign/empire	KINGDOM
CROW	Thorn/whack/rose	BUSH
VEST	Wet/tie/business	SUIT
TRAY	Pepper/mine/shaker	SALT
APPLE	Nucleus/centre/rotten	CORE
CHICK	Bald/screech/emblem	EAGLE
VENOM	Widow/bite/bug	SPIDER
CRATER	Star/saw/hoover	DUST
MASTER	Medal/winner/cup	PRIZE
PICNIC	Orchard/tomato/picker	CHERRY
CHEMIST	Test/cathode/metro	TUBE
PRAIRIE	Pride/trainer/safari	LION
REALITY	Magic/mirage/dream	ILLUSION

**Table:** 

		Set A			Set B		
		Group A	Group B	Group C	Group D	Group E	Group F
Associative relation	Cue & target words	0.05 (0.16)	0.07 (0.16)	0.12 (0.19)	0.02 (0.06)	0.04 (0.09)	0.13 (0.17)
	RAT problems & solutions	0.13 (0.12)	0.15 (0.09)	0.14 (0.10)	0.18 (0.15)	0.15 (0.11)	0.15 (0.08)

Word length	Cue words	5.50 (1.17)	5.50 (1.17)	5.50 (1.17)	5.50 (1.17)	5.58 (1.16)	5.50 (1.17)
	Target words	5.42 (1.24)	5.25 (1.42)	5.42 (1.44)	5.42 (1.08)	5.17 (1.27)	5.25 (1.22)
	RAT problems	7.08 (0.91)	7.22 (0.96)	7.28 (0.80)	7.08 (0.80)	6.97 (1.11)	7.22 (0.77)

Word frequency	Cue words	32.92 (55.02)	39.83 (50.03)	39.67 (83.29)	34.17 (47.70)	40.75 (53.23)	30.58 (33.90)
	Target words	29.08 (19.49)	28.25 (23.53)	29.27 (20.37)	29.25 (22.03)	28.58 (26.28)	28.42 (20.59)
	RAT problems	81.94 (147.21)	78.56 (84.98)	80.86 (133.50)	74.19 (101.04)	70.83 (63.50)	70.22 (50.41)

**Table B1:** Recall accuracies (standard deviations) for criterion tests.

	Baseline	No-think	Baseline versus no-think	
			*t* value	*p*
Experiment 1	90.00 (9.64)	88.33 (11.07)	0.72	0.48
Experiment 2 (Implicit)	86.94 (9.71)	86.67 (11.50)	0.14	0.89
Experiment 2 (Explicit)	85.56 (10.71)	86.11 (11.01)	−0.25	0.81

**Table B2:** Unconditionalized Response Accuracies (Standard Deviations) for RAT Questions.

	Baseline	No-think	Novel	ANOVA	
				F value	*p*
Experiment 1	43.06 (14.53)	37.50 (13.80)	37.96 (12.02)	2.89	0.06
Experiment 2 (Implicit)	49.44 (16.80)	40.00 (17.29)	40.19 (8.28)	6.66	0.01
Experiment 2 (Explicit)	55.56 (16.28)	54.44 (17.47)	42.13 (11.85)	9.41	0.01

**Table C1:** Mean Scores (Standard Deviations, Number of Responses) for Puzzle Solving Questionnaire Items. Note that Q2a, Q2b, and Q3 were answered only by the smaller subset of participants who reported explicit awareness of the link between the RAT and the earlier TNT phase on Q1, or who were unsure of whether they were aware of such a relationship.

	Experiment 1	Experiment 2 (Implicit)	Experiment 2 (Explicit)
Q1	0.50 (0.82)	0.77 (0.86)	1.90 (0.31)
Q2a	3.46 (0.66)	3.25 (0.93)	2.40 (1.19)
Q2b	0.47 (0.78)	1.19 (1.28)	2.30 (0.99)
Q3	2.11 (0.60)	2.00 (0.76)	1.33 (0.48)

## Appendix A 1.

### Materials for word pairs and remote associates test

#### Appendix A 2.

Statistics (Standard Deviations) for Materials in Each Experimental Condition

## Appendix B .

See Table B1, Table B2.

## Appendix C .

See Table C1.

## Footnotes

1 Although the RAT was introduced as a creativity test, recent work suggests it is better characterized as a measure of convergent thinking more related to working memory capacity and IQ (Lee, Huggins, & Therriault, 2014; Chein & Weisberg, 2014; Chuderski & Jastrzębski, 2018), than creativity per se, and that it is weakly related to divergent thinking tasks used to measure creativity. Nevertheless, convergent thinking might also be important to creativity (Lee et al., 2014). For present purposes, although we told participants we were measuring creativity, the status of the RAT as a creativity test is not central to our purpose of measuring indirect influences of experience on thought.

2 Although our sample size was based on convention rather than power analyses – power analyses were conducted retrospectively to ensure that our models were powered appropriately. These analyses were based on the only other study that has used the RAT to examine memory inhibition in implicit memory, though in retrieval-induced forgetting rather than in a retrieval-suppression paradigm (Gomez-Ariza et al., 2017, Experiment 2). Based on their effect size (*f* = 0.40), 26 participants would be needed to obtain 95% power.

3 Bayes Factors were calculated using the Summary Stats add-on for the JASP analysis suite (JASP Team, 2017) and are reported using the convention of *BF*_10_ to reflect a Bayes Factor in support of the alternative hypothesis and *BF_01_* to reflect a Bayes Factor in support of the Null hypothesis. We have conservatively adopted the equivalent of a two-tailed test for each.

4 The power achieved at 30 participants was based on the effect size from the retrieval-induced forgetting paradigm of Gomez-Ariza et al. (2017) is 99.97%. However, retrospectively, using Experiment 1 as a precedent, a sample size of 30 participants in this design would have 85% power to detect suppression-induced forgetting.
